# Self-Assembling RADA16-I Peptide Hydrogel Scaffold Loaded with Tamoxifen for Breast Reconstruction

**DOI:** 10.1155/2017/3656193

**Published:** 2017-06-12

**Authors:** Huimin Wu, Ting Zhou, Lin Tian, Zhengchao Xia, Feng Xu

**Affiliations:** ^1^Fengxian Hospital, Southern Medical University, Shanghai 201400, China; ^2^The Fifth Hospital, Sun Yat-sen University, Zhuhai 519000, China

## Abstract

More and more breast cancer patients prefer autologous fat tissue transfer following lumpectomy to maintain perfect female characteristics. However, the outcome was not satisfactory due to the transplanted fat absorption. In this study, we prepared two RADA16-I peptide scaffolds with and without tamoxifen. Both scaffolds were transparent, porous, and hemisphere-shaped. The hADSCs isolated from liposuction were attached to the scaffold. The growth inhibition of the hADSCs induced by TAM in 2-demensional (2D) culture was higher than that in TAM-loaded hydrogel scaffold 3D culture (*P* < 0.05); however, the same outcomes were not observed in MCF-7 cells. Correspondingly, the apoptosis of the hADSCs induced by TAM was significantly increased in 2D culture compared to that in scaffold 3D culture (*P* < 0.05). Yet the outcomes of the aoptosis in MCF-7 were contrary. Apoptosis-related protein Bcl-2 was involved in the process. In vivo experiments showed that both scaffolds formed a round mass after subcutaneous implantation and it retained its shape after being pressed slightly. The implantation had no effect on the weight and activity of the animals. The results suggested that TAM-loaded RADA16-I hydrogel scaffolds both provide support for hADSCs cells attachment/proliferation and retain cytotoxic effect on MCF-7 cells, which might be a promising therapeutic breast tissue following lumpectomy.

## 1. Introduction

Breast cancer is one of the malignant tumors which threaten the life and health of women [1–3]. Currently there are five treatment tools for breast cancer including surgery, radiation therapy, chemotherapy, hormone therapy, and molecular targeted therapy. Most breast cancer patients first prefer surgery to remove the cancer and then receive postoperative chemotherapy and finally consider breast reconstruction to maintain perfect second female characteristics [4, 5]. Autologous fat tissue transfers once had been used in breast reconstruction. However, the outcome was not satisfactory since the transplanted fat tissue was gradually absorbed [6].

Biocompatible fillers such as collagen, chitosan, and silk have been tried to reconstruct the 3D scaffold for breast tissue [7, 8]. We once constructed polylactide beads loaded with TAM and cultured human mesenchymal stem cells (hMSCs) on the beads [9, 10]. However, it was not convenient to obtain autologous hMSCs from breast cancer patients who underwent surgery. With the great improvement of liposuction and stem cell isolation, the hADSCs are more convenient to get than the hMSCs. The number of hADSCs obtained from liposuction can reach 10^7^ cells per milliliter. Besides, hADSCs also can take part in the revascularization process in the organization repairing [11, 12] and secrete large amounts of cytokines such as VEGF and HGF which have good effect on endothelial cells [13]. Since Zuk et al. [14] first isolated ADSCs from the fatty portion of the liposuction aspirates with collagenase, scientists keep constant struggles on producing safer and more convenient protocols. Yoshimura et al. [15] found out a collagen-free isolation method and it was deemed to be a big progress on practical appliance of ADSCs. Also, RADA16-I peptide (AC-RADARADARADARADA-CONH 2 R, arginine; A, alanine; D, aspartate) is known as a biocompatible material with favorable security. The RADA16-I peptide is ideal to engineer new fillers loaded with anticancer drug and hADSCs both to overcome the short survival of transplanted fat tissue and to kill any remaining cancer cells. In this paper, we fabricated a self-assembling peptide scaffold loaded with TAM aiming to engineer filler biomaterials working as artificial therapeutic breast tissue following lumpectomy.

## 2. Materials and Methods

### 2.1. Hydrogel Scaffold Preparation and Material Characterization

RADA16-I peptide was obtained from Shanghai Jier Biotechnology Co., Shanghai, China. The control hydrogel was prepared by dissolving RADA16-I peptide powder with Milli-Q water (1% v/w) and sonicated for 20 min. TAM-loaded hydrogel was prepared the same but with TAM incorporation. The concentration of TAM in the hydrogel was 25 *μ*mol/L. Both hydrogels were allowed to self-assemble to scaffold overnight.

Surface morphology of the control hydrogel scaffold and TAM-loaded hydrogel scaffold was analyzed by scanning electron microscopy (SEM). Samples were coated with gold, imaged, and photographed by SEM (HITACHI S-3000N, Tokyo, Japan) at an acceleration voltage of 10.00 kV.

Fourier transform infrared (FTIR) spectra of control hydrogel scaffold and TAM-loaded hydrogel scaffold were measured at Analytical and Testing Center, Guangzhou Institute of Chemistry, Chinese Academy of Sciences, according to the general rules for Fourier transform infrared spectrometer (JY/T 001-1996) at the temperature of 20°C and the humidity of 50%. The wavelength range was from 4000 to 400 cm^−1^.

### 2.2. Isolation, Culture, and Identification of hADSCs

The aspirated fat tissue was obtained from the patients undergoing weight-loss liposuction surgery in the hospital. The tissue was washed with phosphate-buffered saline (PBS) twice and digested in PBS containing 0.15% collagenase (Sigma-Aldrich Co., USA) on a shaker at 37°C for 1 hr. The mature adipocytes and connective tissues were removed by centrifugation (800*g*  × 10 min). The pellets were resuspended in medium and passed through a 100 *μ*m mesh then centrifuged again (800*g*  × 10 min). The supernatant was removed and stromal vascular fraction (SVF, containing ADSCs) was obtained. The pellets were resuspended in complete culture medium containing high-glucose Dulbecco's modified Eagle's medium, 10% fetal bovine serum (FBS), 100 U/mL penicillin, 100 *μ*g/mL streptomycin, and 2 mM L-glutamine (all from Gibco Life Technologies Co., USA). The cells were cultured in a humidified atmosphere containing 5% CO_2_ at 37°C. At 3 days after initial plating, the adherent hADSCs were washed twice with PBS to remove the nonadherent cells. After reaching confluence, the hADSCs were trypsinized and identified through membrane receptor phenotyping and differentiation assays. Stem cell markers including CD31, CD34, CD44, CD45, and CD90 were determined by flow cytometry.

### 2.3. Cell Seeding, Growth Inhibition, Apoptosis, and Differentiation Induction

The hADSCs and human breast cancer cell line MCF-7 cells (Type Culture Collection, Chinese Academy of Science, Shanghai, China) were both processed separately in this work. For 2D culture assay, hADSCs and MCF-7 cells were seeded uniformly in 96-well culture plate. For 3D culture assay, there was a nuance. As to hADSCs, first step was putting the hydrogel scaffold into the medium in the 6-well culture plate, then seeding the hADSCs cells slowly from the top of the scaffold so that cells moved and attached to the scaffold, and embedding in the scaffold finally. As to MCF-7 cells, first step was seeding cells in the 6-well culture plate and then setting scaffold into the medium, which simulated the scaffold encountering the remaining cancer cells in vivo.

MCF-7 cells and hADSCs were seeded in culture-plates (2 × 10^4^ cells/well) and were cultured in different conditions separately for 72 h. The growth inhibition rate was evaluated by MTT assay. Apoptosis were measured by flow cytometry (FCM) using Annexin V-FITC Apoptosis Detection Kit (KeyGEN Biotech Co., Jiangsu, China). Briefly, the cells cultured in different conditions as described before were gently harvested with trypsin and washed twice in cold PBS. Annexin V-FITC and PI, 5 *μ*L of each, were added to 500 *μ*L of cell suspension (10^5^ cells/mL) in binding buffer. Samples were left in the dark for 30 min and then analyzed by FCM using a Coulter EPICS XL flow cytometer (LSRForessa, Becton, Dickinson and Company, USA). Data were acquired and analyzed by the FACSDiva (v.6.2) software (Becton, Dickinson and Company, USA).

The hADSCs was induced to adipocyte differentiation by specific differentiation medium, that is, high-glucose DMEM supplemented with 10% FBS, dexamethasone (1 *μ*M), isobutylmethylxanthine (0.5 mM), insulin (10 *μ*g/mL), and indomethacin (100 *μ*M) (all from Sigma, USA) for 25 days.

### 2.4. Proteins Expression by Western Blotting

The treated cells were lysed at ice and collected for protein study. The protein concentration was determined by BCA assay. The proteins were separated by electrophoresis and transferred to a nitrocellulose membrane. The blocked membrane was incubated overnight with primary antibodies against *β*-actin, BAX, or BCL-2 (all antibodies are from Bioworld Technology Inc., Minnesota, USA) at 4°C for above 12 hr. Following washing 3 times with TBST (Tris Buffer Solution + Tween), the membrane was incubated with goat anti-rabbit HRP-conjugated secondary antibodies for 2 hr at room temperature. After being washed 3 times with TBST, the protein was detected using enhanced chemiluminescence reagent (Pierce, Thermo Fisher Scientific, Massachusetts, USA). Images were captured with the Kodak in vivo imaging system F (Eastman Kodak Co., New York, USA). The band intensity was analyzed by ImageJ software (National Institutes of Health, USA). All experiments were performed in triplicate.

### 2.5. Scaffold In Vivo Implantation

Both control hydrogel scaffold and TAM-loaded hydrogel scaffold were seeded with hADSCs in specific differentiation culture medium for 3 days. Then the scaffolds were injected subcutaneously at the second breast pad in female BALB/c-nu mice bilaterally. Control hydrogel scaffold with hADSCs was implanted at one side, while TAM-loaded hydrogel scaffold with hADSCs was implanted on the other side. The mice were all purchased from Laboratory Animal Center of Sun Yet-sen University (Guangzhou, China) and fed in the SPF grade Animal Lab of Southern Medical University (Guangzhou, China).

### 2.6. Statistical Analysis

All data were expressed as mean ± SD, and analysis was performed using SPSS 20.0 for Windows (SPSS Inc., Chicago, IL, USA). One-way analysis of variance (ANOVA) was used for multiple comparisons. A value of *P* < 0.05 was considered to be statistically significant.

## 3. Results

### 3.1. Characterization of Hydrogel Scaffold

**Figure 1:**
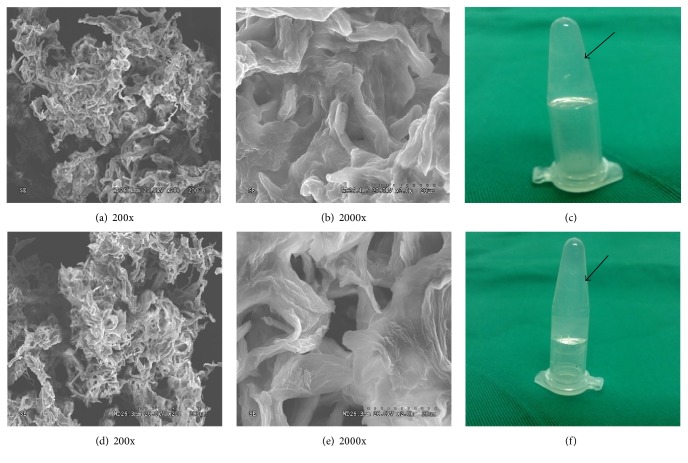
(a) SEM image (200x) of the control hydrogel scaffold; stereoscopic structure can be observed; (b) SEM image (2000x) of the control hydrogel scaffold; the inner space of the scaffold was loose texture and porous. (c) The appearance of the control hydrogel scaffold is colloidal and figurate; (d) SEM images (200x) of the TAM-loaded hydrogel scaffold. The structure of the scaffold is familiar with the control hydrogel scaffold which reveals that TAM has little influence on the framework of the scaffold; (e) SEM images (2000x) of the TAM-loaded hydrogel scaffold; apertures still existed and go deeper than those on control hydrogel scaffold. The change may lead hADSCs to penetrate deeper easily. (f) The appearance of the TAM-loaded hydrogel scaffold; the adjunction of TAM has no effect on the coagulation of the hydrogel scaffold.

As shown in Figure 1, both hydrogel scaffolds were transparent (c, f), porous scaffold with smooth surface and hemisphere-shaped (a, b, d, e). TAM has little effect on the morphology of the peptide hydrogel scaffold. There was no significant characteristics difference between control hydrogel scaffold and TAM-loaded hydrogel scaffold.

**Figure 2:**
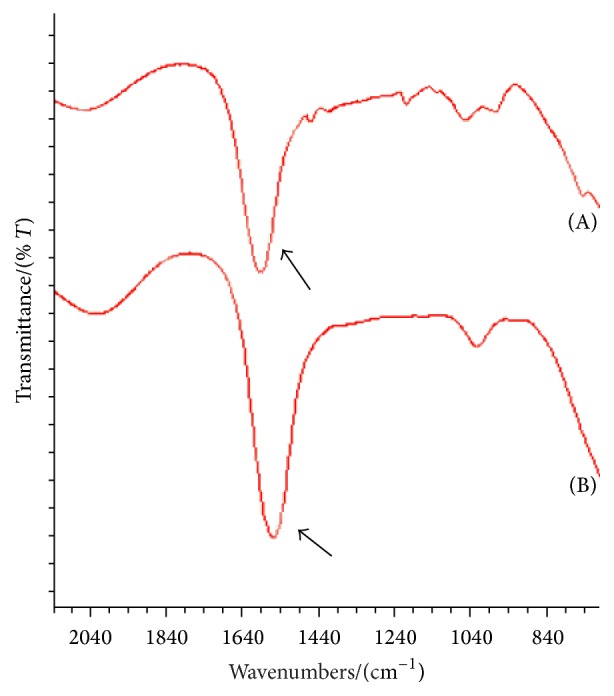
FTIR spectra of 2 hydrogel scaffolds showing amide regions. (A) Control hydrogel scaffold. (B) TAM-loaded hydrogel scaffold.

Comparing with the FTIA of control hydrogel scaffold (Figure 2(A)), changes in the peak heights between 1800 cm^−1^ and 1400 cm^−1^ (amide I and amide II regions) were observed in TAM-loaded hydrogel scaffold (Figure 2(B)). Hydrogen bonds were formed by intermolecular beta sheets and therefore displayed an absorption in the interval 1620 cm^−1^–1640 cm^−1^. Meanwhile unordered structures were present at 1640 cm^−1^–1650 cm^−1^ [16]. An increase near 1640 cm^−1^ peak also occurred, which was due to an increase in unordered structures and random coil features. These conformational changes might be caused by TAM addition.

### 3.2. Identification and Adipogenesis Differentiation of hADSCs

**Figure 3:**
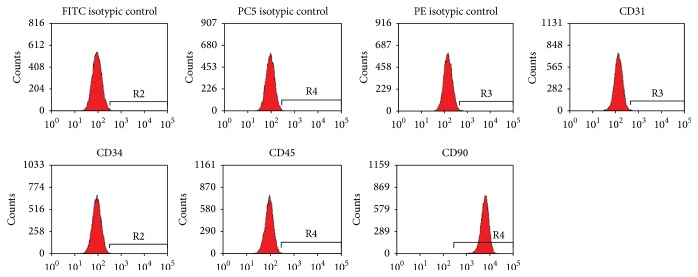
The hADSCs with surface markers: low expression of CD31 (0.17%), CD34 (0.17%), and CD45 (0.15%) and high expression of CD90 (99.94%).

The surface markers expression of hADSCs showed low expression of endothelial marker CD31 (0.17%), haematopoietic marker CD34 (0.17%), and leukocyte marker CD45 (0.15%) but high level of CD90 adhesion marker (99.94%) (Figure 3), which proved the hADSCs were separated successfully.

**Figure 4:**
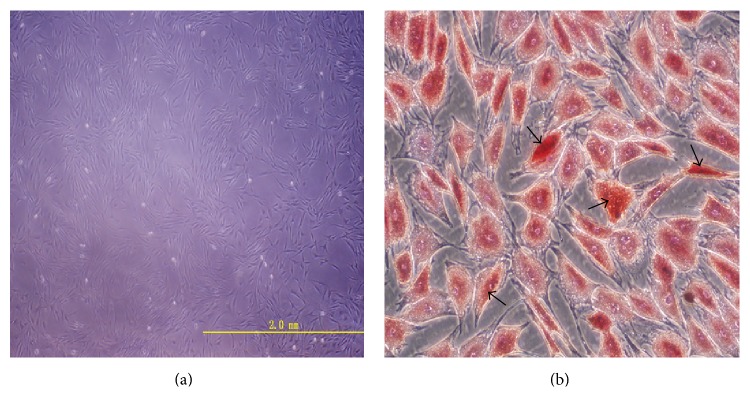
(a) Isolated hADSCs cultured in plate. (b) Differentiation of human ADSCs into adipocytes. Oil red O staining visualizes lipid vacuoles in plate.

After 25 days of adipogenesis differentiation induction, the hADSCs were confirmed to differentiate adipocytes by Oil Red O staining. The adipocytes were also identified morphologically by the presence of lipid vacuoles in the cytoplasm (Figure 4(b)).

### 3.3. Cells Growth Inhibition and Apoptosis

The growth inhibition of the hADSCs induced by TAM in 2D culture was higher than that in TAM-loaded hydrogel scaffold 3D culture (*P* < 0.05). However, the growth inhibition of MCF-7 cells induced by TAM in 2D culture was similar to that in TAM-loaded hydrogel scaffold 3D culture. No significant difference existed between the inhibition rates of TAM on MCF-7 cells in 2D and in 3D culture, but the inhibition rate on hADSCs was significantly reduced from 44.76%  ± 0.04% to 25.36%  ± 0.02% (*P* < 0.01) (Table 1).

**Figure 5:**
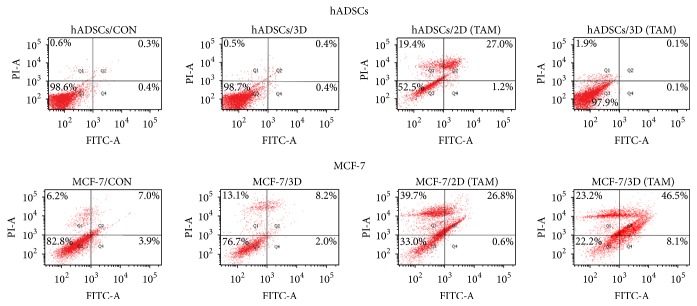
Apoptosis of hADSCs and MCF-7 induced by TAM in different culture environments. The apoptosis of hADSCs induced by TAM was attenuated in 3D environment and it was nearly the same as both 2D and 3D control groups. The apoptosis of MCF-7 induced by TAM was significantly enhanced in 3D environment, even stronger than that in 2D environment. The apoptosis of MCF-7 in 2D and 3D control groups was about the same, so the enhancement should be caused by TAM.

Correspondingly, as shown in Figure 5, the apoptosis of the hADSCs induced by TAM was significantly increased in 2D culture compared to that in scaffold 3D culture (*P* < 0.05) (hADSCs/2D (TAM) versus hADSCs/3D (TAM)). Yet the apoptosis of MCF-7 cells induced by TAM was decreased in 2D culture compared to that in scaffold 3D culture (MCF-7/2D (TAM) versus MCF-7/3D (TAM)). These results may reveal that the 3D environment may enhance the toxicity of TAM on MCF-7 but has less effect on hADSCs.

### 3.4. Apoptosis-Related Proteins Change

**Figure 6:**
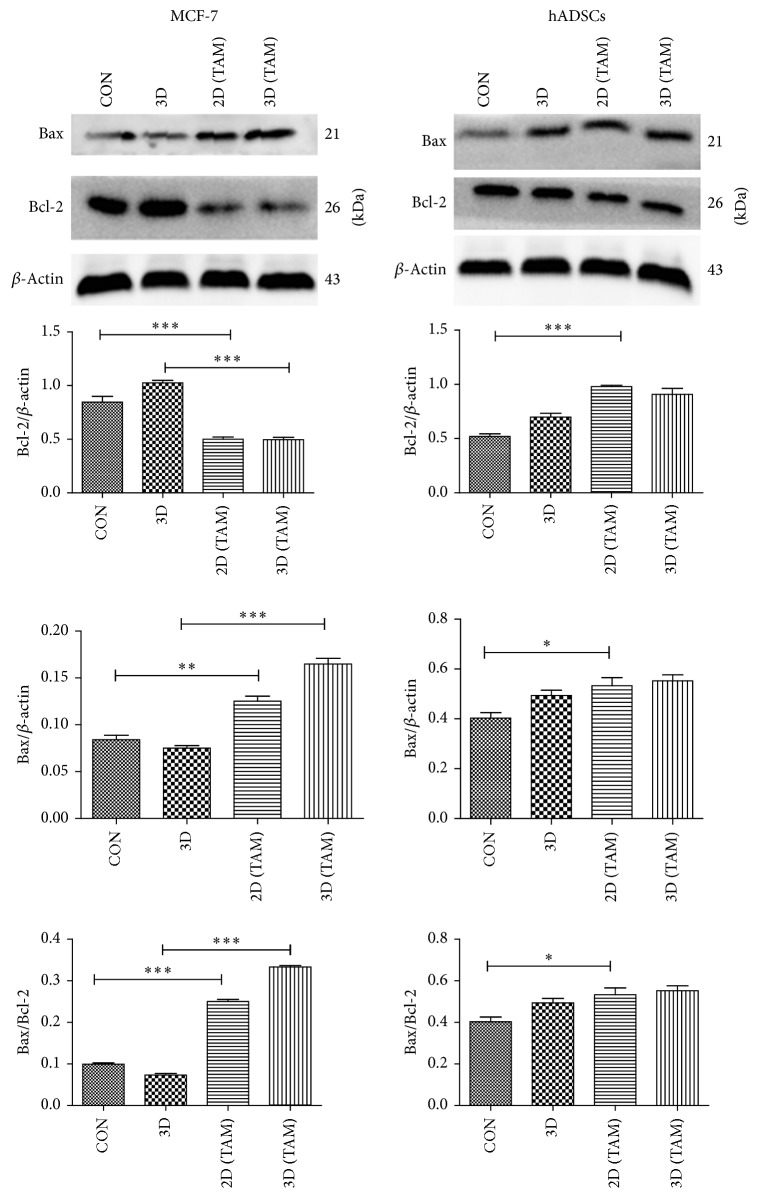
Apoptosis-related proteins analysis of hADSCs and MCF-7 in different groups. Bar graphs show the relative levels of bax and bcl-2 in hADSCs and in MCF-7. Data were presented as mean ± SD of three independent experiments. ^*∗*^*P* < 0.05; ^*∗∗*^*P* < 0.01; ^*∗∗∗*^*P* < 0.001 by one-way analysis of variance.

After 72 hr treatment, the expressions of bcl-2 in the MCF-7 cells in 2D and 3D culture were significantly decreased comparing with the corresponding blank controls (*P* < 0.05), and no significant difference existed between these two groups. The expressions of bcl-2 in the hADSCs in 2D and 3D were nearly the same and also decreased compared to the corresponding blank controls (Figure 6). Bcl-2 is the antiapoptotic protein while bax is the apoptosis promoting protein. This couple of markers can reflect cell apoptosis. The decrease of bcl-2 illustrated that the 3D environment aggravates the apoptosis-inducing effects of TAM on MCF-7. The potentiation is not significant in hADSCs.

### 3.5. Observation of Hydrogel Scaffold Implantation

**Figure 7:**
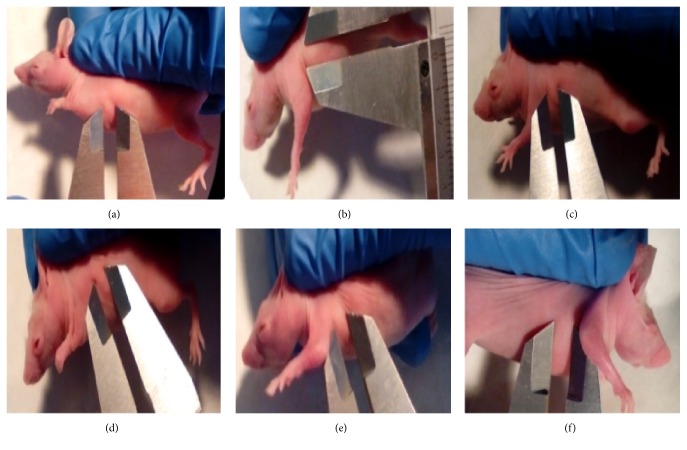
Scaffold implantation for 4 days in nude mice ((a), (b), (c): hydrogel scaffold + hADSCs; (d), (e), (f): hydrogel scaffold + TAM + hADSCs). The formations of hydrogel scaffold were observed in both groups and the shapes are similar.

**Figure 8:**
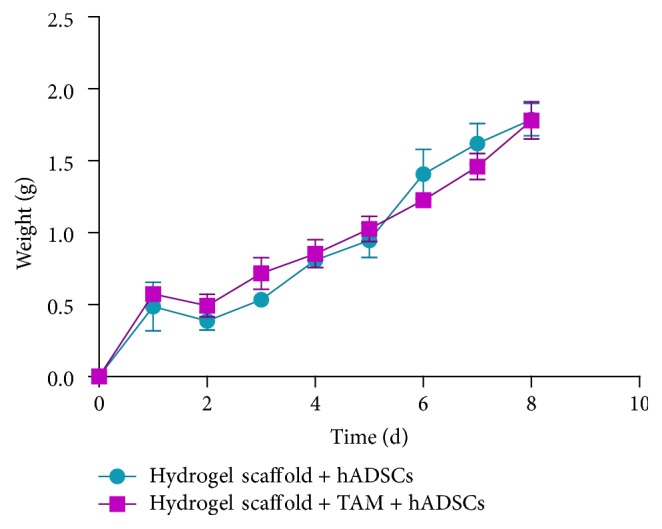
The body weight change of nude mice after subcutaneous implantation. The curves of two groups were about the same.

After subcutaneous injection of hydrogel scaffold with hADSCs or TAM-loaded hydrogel scaffold with hADSCs, respectively, into the nude mice, round mass was formed with regular shape and clear edge and retained its shape after being pressed slightly (Figure 7). As time went on, the mass volume decreased and disappeared at about 7 days (Table 2). No difference occurred between 2 groups on body weight and activity of mice (Figure 8).

## 4. Discussion

Many studies have found that a class of self-assembling peptides provides an ideal and simplified extracellular matrix material which might be used for tissue engineering and regenerative medicine. The application of self-assembling peptide in hydrogel scaffold has been proven useful in several cell culture systems due to its excellent biocompatibility, biodegradability, and low risk of biological contamination [16–27]. Such scaffolds could establish a growth environment to simulate that in vivo [28]. RADA16-I (AcN-RADARADARADARADA-CNH), composed of natural amino acids, is a kind of injectable self-assembled polypeptide which can form a hydrogel scaffold with a large number of nanofibers under certain conditions. The structure of RADA16-I hydrogel scaffold was similar to the extracellular matrix, which could provide a good culture condition for the cells. The hydrogel not only supports the adhesion and growth of cells but also is conducive to the exchange of information and material between cells [29, 30]. It has been found that self-assembled peptide hydrogel can provide excellent microenvironment for the growth and differentiation of precursor cells and stem cells [31]. In addition, the RADA16-I peptide hydrogel has a superior biocompatibility and can be designed according to the actual application needs. These characteristics are of benefit to build individual materials to meet the needs of the use. The hydrogel contains higher than 99% of water, which is suitable for carrying drugs or therapeutic proteins. If it is needed to prepare a special drug loading system, it can also be achieved by changing the composition of the peptide. Furthermore, this material can be tailor-made. Various bioactive short peptide motifs that act as analogues of soluble growth factors could be easily conjugated to the C-terminus of RADA16-I to maintain both the bioactivity and biosafety. At present, the RADA16-I hydrogel has been applied to the regeneration of bone, muscle, nerve, blood vessel, and so on and has received wide concern [23, 32, 33].

The choice of seed cell plays an important role in tissue engineering. Stem cells not only have the ability of self-replication but also can be differentiated into a variety of functional cells under certain conditions. Compared to stem cells of other sources, hADSCs from fat tissue are relatively easy to obtain in clinical practice. In this work, we separated hADSCs from fat tissue which was obtained from patients with weight-loss liposuction surgery. The cell surface protein markers showed that the separated cells had a high expression of CD_90_ but low expression of CD_31_, CD_34_, and CD_45_, which confirmed them as stem cells [34, 35].

As we all know, tamoxifen, one of the selective estrogen receptor modulators, has the longest track record in breast cancer and is approved in the United States for risk reduction in high-risk women and in the management of advanced disease. Data from the 2005 EBCTCG (Early Breast Cancer Trial Collaboration Group) showed that 5 years of adjuvant tamoxifen for ER-positive disease reduced the annual breast cancer death rate by 31% regardless of age or chemotherapy use. Duration of tamoxifen therapy (5 years versus 2 years) is important, and the annual breast cancer mortality rates are similar during years 0 to 4 and 5 to 14, with a cumulative reduction in mortality twice as large at 15 years as after 5 years since diagnosis [36]. Some data support the use of adjuvant tamoxifen for 5 years, but two large, ongoing international trials (Adjuvant Tamoxifen Long versus Short [ATLAS] and Adjuvant Tamoxifen Treatment, Offer More? [aTTom]) are examining longer durations of therapy and a first report from the ATLAS suggests a benefit for 10 years of tamoxifen [37]. But long-term medication will be a big challenge on drug concordance and drug delivery system may resolve this problem properly.

Based on our pilot work and others' work, we chose 25 *μ*mol/L of TAM both in 2D culture and in 3D hydrogel scaffold [38]. We found that the growth inhibitions of TAM on MCF-7 and hADSCs in different culture environments were different. AS to hADSCs, TAM-loaded scaffold in 3D culture weakened the effect of TAM compared to that in 2D culture, which suggested that the hydrogel scaffold may benefit proliferation of hADSCs which were embedded inside the scaffold. The scaffold in 3D culture has somewhat protection for hADSCs from TAM. The growth inhibition of MCF-7 cells in TAM-loaded hydrogel scaffold in 3D culture had no significant difference with that in 2D culture. We surmised that TAM released from the scaffold is the same as in 2D culture to inhibit the cancer cells. The scaffold in 3D culture had no beneficial effects for MCF-7 cells from TAM inhibition.

Tamoxifen inhibited breast cancer cell via inducing apoptosis [39]. In this work, the apoptosis rate of MCF-7 in 2D culture and 3D culture was 27.4% and 56.6%, respectively, which were higher than hADSCs in 2D culture (13.4%) and 3D culture (1.6%). We conjectured that MCF-7 cells had more estrogen receptors (ER) than hADSCs, displayed as cytotoxicity difference on two kinds of cells. The decrease of bcl-2 expression of MCF-7 cells in 3D culture also suggested that hydrogel scaffold did not weaken cell apoptosis induced by TAM.

Animal experiments showed that both implants (hydrogel scaffold + TAM + hADSCs, hydrogel scaffold + hADSCs) could form a round mass that looks like the shape of breast. Though the implants were maintained in vivo just up to 7 days, the degradation and absorption did not irritate or cause inflammation. These results suggested that the implant has good biocompatibility, biodegradability, and low risk of biological contamination. The maintaining time of both implants was similar, which revealed that the addition of TAM did not influence the degradation of hydrogel scaffold. More importantly, the existence of the round mass has little effect on the weight change and the survival of nude mice, which demonstrated the nontoxic characteristics. How to prolong the maintaining time of our engineering artificial therapeutic breast tissue is worth studying further in future.

From the existing researches, the applications for self-assembled peptide hydrogel in 3D environment simulation and drug delivery call for optimism [40]; however, few researchers combine these two into one. We made a preliminary study on this assumption and the results are desirable and feasible. In order to further optimize the practicability of the combination, the degradation and absorption rate of the peptide scaffold, also the solution rate of the loaded drug, should play an important role.

## 5. Conclusion

Tamoxifen-loaded RADA16-I peptide hydrogel scaffold with hADSCs may be used for artificial therapeutic breast tissue following lumpectomy. But the long-term persistence of in vivo engineering tissue should be the major challenge, and further research on the mechanism of how the 3D environment affects pesticide effect is also in need. Moreover, the drug delivery system requires the balance of maintaining an effective therapeutic dose in a desired location, managing and limiting side effects, and patient compliance [41]. Though our work predicts that the combination of tissue engineering and drug delivery system is prospective, these challenges are worth being discussed in depth.

## Figures and Tables

**Table 1:** The growth inhibition of tamoxifen (TAM) to cells in different environments.

Cells	Culture environment	Growth inhibition rate (*x* ± *s*)
hADSCs	TAM in 2D culture	44.76% ± 4.4%
hADSCs	TAM-loaded scaffold 3D culture	25.36% ± 2.1%^*∗*^
MCF-7	TAM in 2D culture	80.25% ± 2.5%
MCF-7	TAM-loaded scaffold 3D culture	81.86% ± 2.8%

^*∗*^ *P* < 0.05 compared to corresponding 2D culture.

**Table 2:** The size of two hydrogel scaffold complex implantations.

Time (d)	Volume of implantation ($\overset{-}{\chi }\pm s$) (mm^3^)					
	Hydrogel scaffold + hADSCs group			Hydrogel scaffold + TAM + hADSCs group		
0	—	—	—	—	—	—
1	87.5 ± 2.2	99.2 ± 2.5	50.2 ± 2.2	53.0 ± 2.5	140.9 ± 2.5	90.6 ± 2.8
2	55.3 ± 2.4	56.3 ± 2.4	36.8 ± 2.3	43.5 ± 2.2	55.0 ± 2.2	42.4 ± 2.5
3	32.0 ± 2.1	28.7 ± 2.2	27.4 ± 2.3	35.6 ± 2.1	31.4 ± 2.3	28.1 ± 2.5
4	22.1 ± 2.2	18.8 ± 2.3	18.0 ± 2.4	20.0 ± 2.4	13.3 ± 2.2	24.8 ± 2.4
5	19.1 ± 2.3	12.9 ± 2.2	15.4 ± 2.1	12.5 ± 2.2	4.0 ± 2.6	19.2 ± 2.7
6	8.9 ± 2.3	6.5 ± 2.1	6.78 ± 2.5	4.6 ± 2.5	—	11.8 ± 2.3
7	6.7 ± 2.1	—	—	—	—	7.84 ± 2.6
8	—	—	—	—	—	—
